# Retraction: Net Expression Inhibits the Growth of Pancreatic Ductal Adenocarcinoma Cell PL45 *In Vitro* and *In Vivo*

**DOI:** 10.1371/journal.pone.0315984

**Published:** 2024-12-12

**Authors:** The PLOS ONE Editors

Following the publication of this article [1], concerns were raised regarding Figs 2 and 4. Specifically,

- The Control and Ad5/F35-GFP panels appear to partially overlap in the following:
  ○ Fig 2C
  ○ Fig 4D, c-fos panels
  ○ Fig 4D, p21 panels
  ○ Fig 4D, TUNEL panels
- In Fig 2E Cyclin E, c-Jun and β-actin western blot panels, there appear to be irregularities in the background.

The corresponding author (XM) and first author acknowledged that errors occurred in the preparation of Figs 2C and 4D. They provided revised figures and provided some original data but stated that not all of the original data could be located.

The authors stated that the original uncropped images are not available for the western blots presented in Fig. 2E, and they acknowledged that the indicated panels were edited to remove contaminated areas.

The *PLOS ONE* Editors retract this article due to the above unresolved concerns which question the integrity and reliability of the reported results.

BL agreed with the retraction. XM did not respond to the final editorial decision. XW, QZ, LLi, YZ, DH, YQ, LLu, and XW either did not respond directly or could not be reached.
